# Validation of a Novel *Fgf10*^*Cre*–*ERT*2^ Knock-in Mouse Line Targeting FGF10^Pos^ Cells Postnatally

**DOI:** 10.3389/fcell.2021.671841

**Published:** 2021-05-13

**Authors:** Xuran Chu, Sara Taghizadeh, Ana Ivonne Vazquez-Armendariz, Susanne Herold, Lei Chong, Chengshui Chen, Jin-San Zhang, Elie El Agha, Saverio Bellusci

**Affiliations:** ^1^School of Pharmaceutical Sciences, Wenzhou Medical University, Wenzhou, China; ^2^Key Laboratory of Interventional Pulmonology of Zhejiang Province, Department of Pulmonary and Critical Care Medicine, The First Affiliated Hospital of Wenzhou Medical University, Wenzhou, China; ^3^Department of Internal Medicine, Universities of Giessen and Marburg Lung Center (UGMLC), Cardio-Pulmonary Institute (CPI), Member of the German Center for Lung Research (DZL), Justus-Liebig University Giessen, Giessen, Germany; ^4^Institute for Lung Health (ILH), Giessen, Germany; ^5^National Key Clinical Specialty of Pediatric Respiratory Medicine, Discipline of Pediatric Respiratory Medicine, Institute of Pediatrics, The Second Affiliated Hospital of Wenzhou Medical University, Wenzhou, China

**Keywords:** *Fgf10*, knock-in Cre line, lipofibroblast, adult lung, lineage tracing

## Abstract

*Fgf10* is a key gene during development, homeostasis and repair after injury. We previously reported a knock-in *Fgf10*^*Cre*–*ERT*2^ line (with the Cre-ERT2 cassette inserted in frame with the start codon of exon 1), called thereafter *Fgf10*^*Ki*–*v*1^, to target FGF10^Pos^ cells. While this line allowed fairly efficient and specific labeling of FGF10^Pos^ cells during the embryonic stage, it failed to target these cells after birth, particularly in the postnatal lung, which has been the focus of our research. We report here the generation and validation of a new knock-in *Fgf10*^*Cre*–*ERT*2^ line (called thereafter *Fgf10*^*Ki*–*v*2^) with the insertion of the expression cassette in frame with the stop codon of exon 3. *Fgf10*^*Ki*−*v*2/+^ heterozygous mice exhibited comparable *Fgf10* expression levels to wild type animals. However, a mismatch between *Fgf10* and *Cre* expression levels was observed in *Fgf10*^*Ki*–*v*2/+^ lungs. In addition, lung and limb agenesis were observed in homozygous embryos suggesting a loss of *Fgf10* functional allele in *Fgf10*^*Ki*–*v*2^ mice. Bioinformatic analysis shows that the 3′UTR, where the Cre-ERT2 cassette is inserted, contains numerous putative transcription factor binding sites. By crossing this line with tdTomato reporter line, we demonstrated that tdTomato expression faithfully recapitulated *Fgf10* expression during development. Importantly, *Fgf10*^*Ki*–*v*2^ mouse is capable of significantly targeting FGF10^Pos^ cells in the adult lung. Therefore, despite the aforementioned limitations, this new *Fgf10*^*Ki*–*v*2^ line opens the way for future mechanistic experiments involving the postnatal lung.

## Introduction

The fibroblast growth factor (FGF) family consisting of 22 members is divided into three groups: the paracrine FGF group signaling through FGFR and heparin-sulfate proteoglycans, the endocrine FGF group signaling through FGFR with Klotho family of proteins as co-receptors, and the intracellular FGF group involved in FGFR independent signaling (Ornitz and Itoh, 2001). The FGF7 subgroup which contains FGF3, 7, 10, 22 belongs to the paracrine FGF group. These growth factors interact mostly with the FGFR2b receptor. FGF10 in particular has been shown to play important roles during development, homeostasis and repair after injury (Yuan et al., 2018). In the lung, it plays a crucial role in regulating branching morphogenesis (Jones et al., 2020). Genetic deletion of either *Fgf10* or its predominant receptor *Fgfr2b* leads to agenesis of both the limb and the lung, specific portions of the gut, the pancreas as well as the mammary, lacrimal and salivary glands (Min et al., 1998; Sekine et al., 1999; Ohuchi et al., 2000; Mailleux et al., 2002; Jaskoll et al., 2005; Parsa et al., 2008). During homeostasis, Fgfr2b signaling has been shown to be critical for the regeneration of the incisors in mice as well as for the maintenance of the terminal end buds in the mammary gland (Parsa et al., 2008, 2010). Lineage tracing of FGF10^Pos^ during development indicated that these cells serve as progenitors for lipofibroblast as well as vascular and airway smooth muscle cells (El Agha et al., 2014).

In the context of the repair process, *Fgf10* deletion in peribronchial mesenchymal cells leads to impaired repair following injury to the bronchial epithelium using naphthalene (Volckaert et al., 2011; Moiseenko et al., 2020). On the other hand, overexpression of *Fgf10* reduces the severity of lung fibrosis in bleomycin-induced mice (Gupte et al., 2009). Given these diverse biological activities, it is important to generate and validate mouse knock-in lines allowing to monitor the localization, fate and status of FGF10^Pos^ cells during development, homeostasis and repair after injury.

We have previously generated a *Fgf10*^*Cre*–*ERT*2^ knock-in mouse line, called thereafter *Fgf10*^*KI*–*v*1^ mice, to monitor the fate of FGF10^Pos^ cells after tamoxifen (Tam) administration (El Agha et al., 2012). In these mice, the Tam-inducible Cre recombinase (Cre-ERT2-IRES-YFP) was inserted in frame with the start codon of the endogenous *Fgf10* gene. *Fgf10*^*KI*–*v*1^ corresponds to a loss-of-function allele for *Fgf10* as evidenced by our observation that *Fgf10*^*KI*–*v*1/*KI*–*v*1^ homozygous embryos die at birth from multi-organ agenesis, including the lung. In the *Fgf10^*KI*–*v*1/+^* lungs, the expression of *Cre* gradually decreases to almost undetectable levels postnatally, rendering the monitoring of FGF10^Pos^ cells postnatally impossible. This is likely due to the deletion of intronic sequences containing key transcription factor binding sites at the insertion site of the Cre-expression cassette.

In order to circumvent this problem, we therefore generated a new Cre-ERT2 knock-in line (named *Fgf10*^*KI*–*v*2^) by targeting the 3′UTR of the endogenous *Fgf10* gene. We here provide experimental evidence for the validation of these mice. Besides a PCR-based strategy to genotype the *Fgf10*^*KI*–*v*2^ allele, we have also established a qPCR-based approach to monitor the expression levels of *Fgf10* and *Cre* at different developmental stages in the lung. *Fgf10*^*KI*–*v*2/*KI*–*v*2^ homozygous embryos have been generated to check for developmental defects. *Fgf10^*KI*–*v*2/+^* lines were crossed with the *tdTomato*^*flox*^ mice to validate, at two distinct embryonic stages, the expression patterns of tdTomato in previously known domains of *Fgf10* expression. Importantly, we validated the use of these mice in the adult stages to target FGF10^Pos^ cells in the lung. Flow cytometry analysis and immunofluorescence staining were carried out to further characterize the contribution of these cells to the lipofibroblast lineage. Bioinformatic analysis of the insertion site of the Cre-ERT2 cassette in the 3′UTR was also carried out. Altogether, our results indicate that the new *Fgf10*^*Cre*–*ERT*2^ line can be successfully used to target FGF10^Pos^ cells both in embryonic and adult stages.

## Materials and Methods

### Genotyping

Two pairs of primers were used to determine the genotype of *Fgf10*^*Ki*–*v*2^ knock-in mice. Primer P1 (5′-AACACC TCTGCTCACTTCCTC-3′); and primer P2 (5′-AGGGTCCACC TTCCGCTTTT-3′) were used to detect the knock-in allele (252 bp band) whereas primer P3 (5′-GCAGGCAAA TGTATGTGGCA-3′) and primer P4 (5′-TGCTTGCGTGTCT TACTGCT-3′) were used to detect the wild-type allele (580 bp band). The PCR program consists of a denaturation step at 94°C for 3 min, followed by 34 cycles of denaturation (94°C for 1 min), annealing (60°C for 30 s) and extension steps (72°C for 300 s). The program ends with a completion step at 4°C for infinity hold. Each PCR tube contains 4.3 μL of H_2_O, Taq DNA Polymerase in 5.5 μL of Qiagen Master Mix (QIAGEN, Hilden, Germany), 10 pmol of each primer, and 50 ng of genomic DNA in a final volume of 11 μL.

### Mice and Tamoxifen Administration

All mice were kept under specific pathogen free (SPF) conditions with unlimited food and water. *tdTomato*^*flox*/*flox*^ reporter mice were purchased from Jackson lab (B6; 129S6-Gt(ROSA)^26*Sortm*9(*CAG*–*tdTomato*)*Hze*^/J, ref 007905). Embryonic day 0.5 (E0.5) was assigned to the day when a vaginal plug was detected. Animal experiments were approved by the Regierungspraesidium Giessen (approval number RP GI20/10-Nr. G47/2019). Tamoxifen stock solution was prepared by dissolving tamoxifen powder (Sigma, T5648-5G) in corn oil at a concentration of 20 mg/mL at room temperature and stored in −20°C. Adult mice received 3 successive intraperoneal (IP) injections of tamoxifen (0.25 mg/g body weight) before analysis. Pregnant mice received a single IP injection of tamoxifen (0.1 mg/g body weight) and pups also received a single subcutaneous injection of tamoxifen (0.2 mg/pup) before analysis. Dissected mice were examined using Leica M205 FA fluorescent stereoscope (Leica, Wetzlar, Germany) and images were acquired using Leica DFC360 FX camera. Figures were assembled in Adobe Photoshop and Illustrator.

### Quantitative Real-Time PCR and Statistical Analysis

Freshly isolated embryos and lungs were lysed, and RNA was extracted using RNeasy kit (74106, Qiagen, Hilden, Germany). One microgram of RNA was used for cDNA synthesis using Quantitect Reverse Transcription kit (205311, Qiagen). Primers and probes for *Fgf10*, *Cre*, and β*2-Microglobulin* (*B2M*) were designed using NCBI Primer-BLAST^1^. More details about the used primers and probes can be found in Supplementary Table 1. Quantitative real-time PCR (qPCR) was performed using LightCycler 480 real-time PCR machine (Roche Applied Science). Samples were run in doublets using *B2M* as a reference gene and the delta Ct method was used to calculate the relative quantification. GraphPad Prism 7.0 software was used to generate and analyze data. Statistical analyses were performed using Student’s *t*-test (for comparing two groups) or One-way ANOVA (for comparing three or more groups). Data were considered significant if *P* < 0.05.

### Flow Cytometry

Freshly dissected lung were washed with Hanks’ balanced salt solution (HBSS, 14175-095, Thermo Fisher) and kept on ice. Sharp blades were used to cut the lung into small pieces and digested with 0.5% collagenase Type IV in HBSS (17104019, Life Technologies, Invitrogen) for 45 min at 37°C. Lung homogenates were then passed through 18, 21, and 24G needles followed by 70 and 40 μm cell strainers (542070 and 542040, Greiner Bio-one International). Lung homogenates were centrifuged at 200g at 4°C for 10 min and cell pellets were then re-suspended in HBSS. 20 (μL of sample is taken as an unstained control. Antibodies against CD45 (103114, APC-conjugated; 1:50), CD31 (102409, APC-conjugated; 1:50), EPCAM (APC-Cy7-conjugated; 1:50) and SCA1 (108120, Pacific blue-conjugated; 1:50) (all from Biolegend) as well as LipidTOX stain (FITC-conjugated, 1:200) (H34350, Life Technologies, Invitrogen) were applied for 30 min on ice in the dark. Samples were then washed with 1 mL of HBSS at 4°C for 5 min. FACSAria III cell sorter (BD Biosciences) was used to carry out the FACS measurements and sorting. Endogenous tdTomato signal was detected through PE channel. Gates were set up according to the unstained controls.

### Bioinformatics

National Center of Biotechnology Information (https://www.ncbi.nlm.nih.gov/gene/) and rVista (rVista; http://rvista.dcode.org) were used to find the murine *Fgf10* sequence and the identification of putative transcription factor binding site (TFBS) was done by using PROMO software^2^. The list of putative TFBS located in the area including exon 3 and 3′UTR was further compared with previously identified transcription factors expressed in the lung mesenchyme (Herriges et al., 2012).

## Results

### Generation of a Novel *Fgf10* Knock-in Line (*C57BL6-Fgf10^*tm*2(*YFP*–*Cre*–*ERT*2)*Sbel*^/J* Aka *Fgf10*^*KI*–*v*2^) With the Insertion of the Cre-ERT2 Cassette in Frame With the Stop Codon of Exon3

**FIGURE 1:**
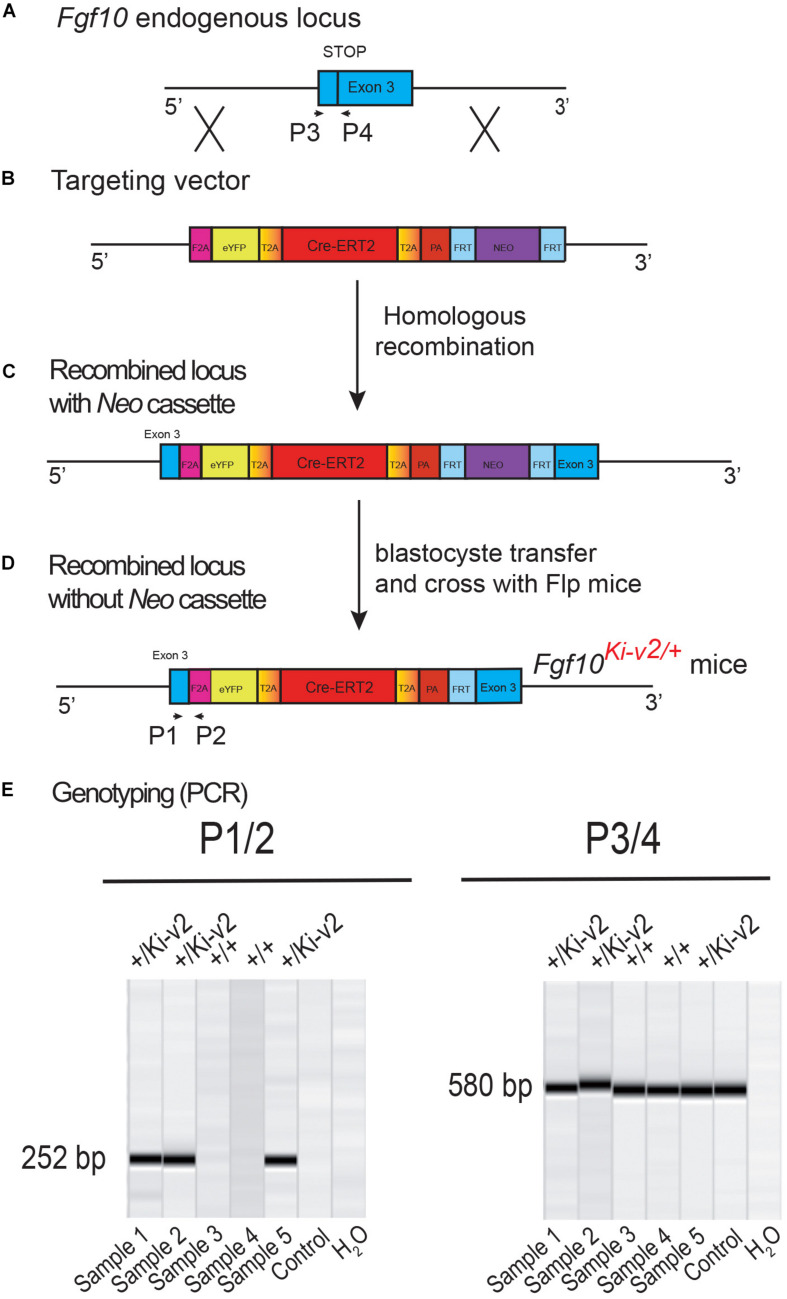
Generation and genotyping of the novel *Fgf10*^*Ki-v*2^ line. **(A,B)** Homologous recombination was carried out to insert the *F2A-eYFP-T2A-Cre-ERT2- T2A-PA-NEO* construct in frame with the stop codon of exon 3 of the mouse *Fgf10* gene. Neomycin resistance coding gene was used for the positive selection. **(C,D)** Recombined ES cell clones were treated with flipase to remove the Neo cassette and blastocyste transfer of the selected ES cells was carried out to generate chimera animals. **(E)** PCR strategy to genotype mutant and wild type animals. Primers 1 and 2 were used for the detection of the mutant *Fgf10*^*Ki-v*2^ allele (252 bp) and Primers 3 and 4 were used for the detection of the wild type *Fgf10*^+^ allele (580 bp).

129Sv ES cells were electroporated with a targeting vector containing the first 3 kb of exon 3 of the *Fgf10* open reading frame (Figures 1A,B). Immediately downstream of the stop codon of exon 3 is the F2A sequence encoding for the self-cleaving peptide, followed by the coding sequence of eYFP, the self-cleaving peptide sequence T2A, the tamoxifen-inducible form of Cre recombinase (Cre-ERT2) (Feil et al., 1997), and the Neomycin-resistance gene (Neo), respectively. Resistant ES cell clones were selected, screened by PCR and then verified by Southern blotting. Selected ES clones were injected into C57BL/6J blastocysts to generate chimeric pups (Figure 1C). Chimeras were then crossed with C57BL/6J mice ubiquitously expressing Flp recombinase to generate heterozygous *Fgf10*^*Ki*–*v*2^ knock-in mice where the Neo cassette was totally excised (Figure 1D). Genotyping strategy with primers P1/P2 (with P1 located just before the STOP codon and P2 being part of the F2A sequence) to detect the *Fgf10*^*Ki*–*v*2^ mutant allele and P3/P4 (located before and after the STOP codon in exon 3, respectively) to detect the *Fgf10*^+^ wild type allele (Figure 1E).

### *Fgf10*^*Ki*–*v*2^ Is a Loss-of-Function Allele

**FIGURE 2:**
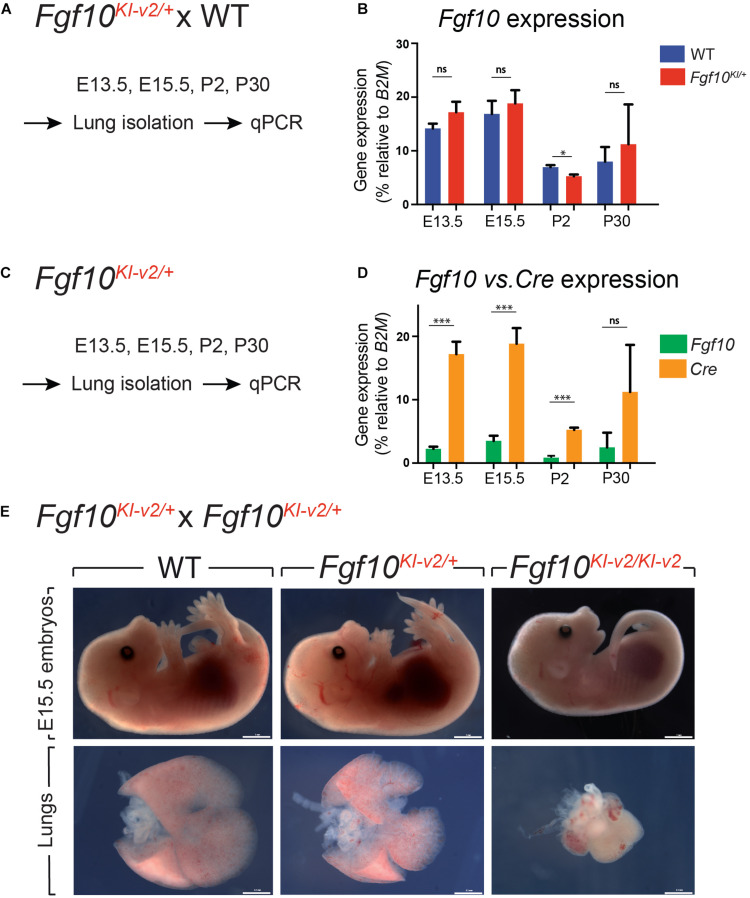
*Fgf10*^*Ki-v*2^ is a loss-of-function allele. **(A)** *Fgf10^*Ki-v*2/+^* and *Fgf10^+/+^* mice were crossed and lungs were isolated at E13.5, E15.5, P2, and P30 for qPCR analysis. **(B)** qPCR results for *Fgf10* expression in *Fgf10^+/+^* and *Fgf10^*Ki-v*2/+^* at these time points. **(C,D)** Lungs were isolated from *Fgf10^*Ki-v*2/+^* at different time points and the expression levels of *Fgf10* and *Cre* were analyzed by qPCR. **(E)** *Fgf10^*Ki-v*2/+^* animals were crossed to generate *Fgf10^+/+^*, *Fgf10^*Ki-v*2/+^*, and *Fgf10*^*Ki-v*2/*Ki-v*2^ embryos at E13.5. Note the absence of limbs and lungs in the *Fgf10*^*Ki-v*2/*Ki-v*2^ embryos. Scale bar in **(E)**: whole embryo: 1 mm; dissected lungs: 0.1 mm. *P ≤ 0.05, ***P ≤ 0.001.

Our initial design of the novel *Fgf10*^*Ki*–*v*2^ knock-in line targeting the 3′UTR was conceived to allow normal expression of *Fgf10*. We carried out the initial validation for *Fgf10* expression in *Fgf10*^*Ki*–*v*2/+^ vs. *Fgf10^+/+^* (WT) in the lung of embryonic and postnatal mice isolated at different time-points (Figure 2A). Our results indicated that *Fgf10* expression level in *Fgf10*^*Ki*–*v*2/+^ lungs is comparable to the one observed in the *Fgf10^+/+^* lungs at all these time-points (Figure 2B). Next, we compared *Fgf10* vs. *Cre* expression in *Fgf10*^*Ki*–*v*2/+^ lungs at different time-points (Figure 2C). Our results indicate a lower level of *Cre* compared to *Fgf10* at all these time-points (Figure 2D). This difference between *Cre* and *Fgf10* expression in *Fgf10*^*Ki*–*v*2/+^ lungs suggests that the insertion of the Cre-ERT2 cassette in the 3′UTR disrupted the expression of the endogenous *Fgf10* gene produced from the recombined allele. Together with *Fgf10* expression in *Fgf10*^*Ki*–*v*2/+^ vs. *Fgf10^+/+^* (WT), this result suggests that *Fgf10* expression from the non-recombined allele in *Fgf10*^*Ki*–*v*2/+^ lungs is increased to compensate the loss of *Fgf10* expression from the recombined allele.

To determine whether the insertion of Cre-ERT2 in the endogenous *Fgf10* locus led to loss of function of *Fgf10*, *Fgf10*^*Ki*–*v*2^ heterozygous animals were self-crossed and embryos were harvested at E15.5. *Fgf10*^*Ki*–*v*2/*Ki*–*v*2^ homozygous embryos suffered from lung and limb agenesis, which is consistent with complete loss of function of *Fgf10* (Figure 2E). Analysis of *Fgf10* expression by qPCR at that stage indicated a drastic reduction in *Fgf10* expression in *Fgf10*^*Ki*–*v*2/*Ki*–*v*2^ embryo (*n* = 1) compared to *Fgf10*^*Ki*–*v*2/+^ or WT lungs (Supplementary Figure 1). We therefore conclude that the *Fgf10*^*Ki*–*v*2^ allele corresponds to a *Fgf10* loss-of-function allele.

### Validation of Cre Activity to Label FGF10^Pos^ Cells During Embryonic Development

**FIGURE 3:**
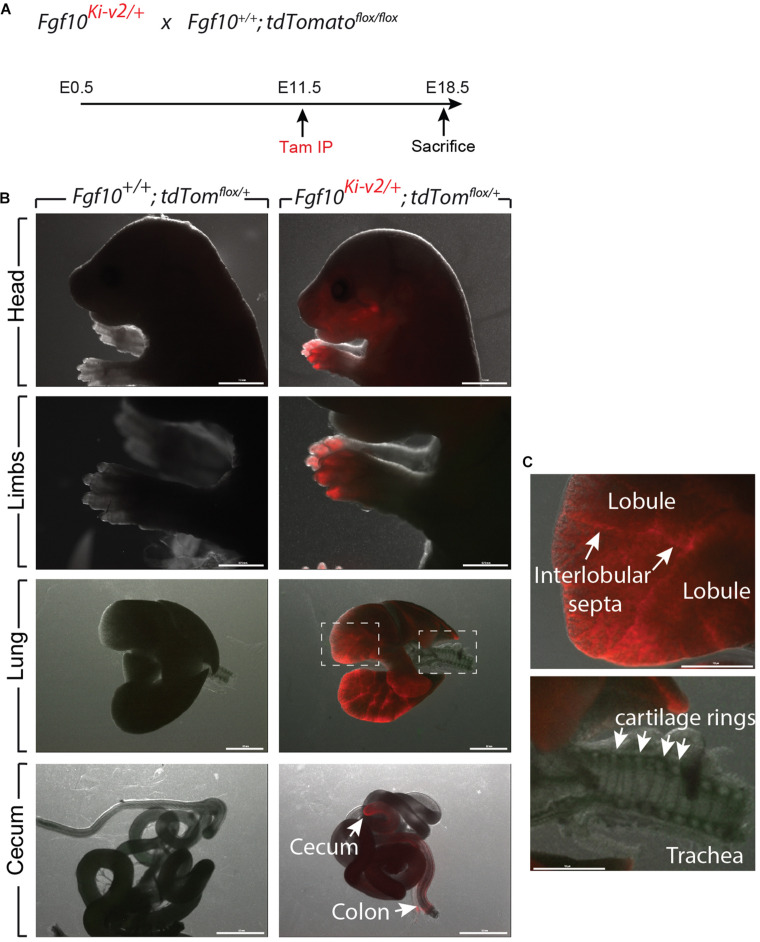
Validation of the labeling of FGF10^Pos^ cells at E11.5. **(A)** *Fgf10^*Ki-v*2/+^* were crossed with *Fgf10^+/+^*; *tdTom*^*flox*/*flox*^ mice. Pregnant females received a single IP injection of tamoxifen when the embryos were at E11.5 and sacrificed at E18.5. **(B)** Head, limbs, lung and cecum of *Fgf10^+/+^*; *tdTom^*flox*/+^* and *Fgf10^*Ki-v*2/+^*; *tdTom^*flox*/+^* embryos are shown. Note the absence of fluorescence in the *Fgf10^+/+^; tdTom^*flox*/+^* indicating that the non-recombined *LoxP-Stop-LoxP-tdTomato* allele is not leaky. **(C)** Higher magnification of lung and trachea showing enriched tdTomato expression in the interlobular septa and the lack of tdTomato expression between the cartilage rings, respectively. Scale bar in **(B)**: head: 1.5 mm, Limb: 0.75 mm, Lung: 0.5 mm, cecum: 0.5 mm. Scale bar in **(C)**: 125 μm.

**FIGURE 4:**
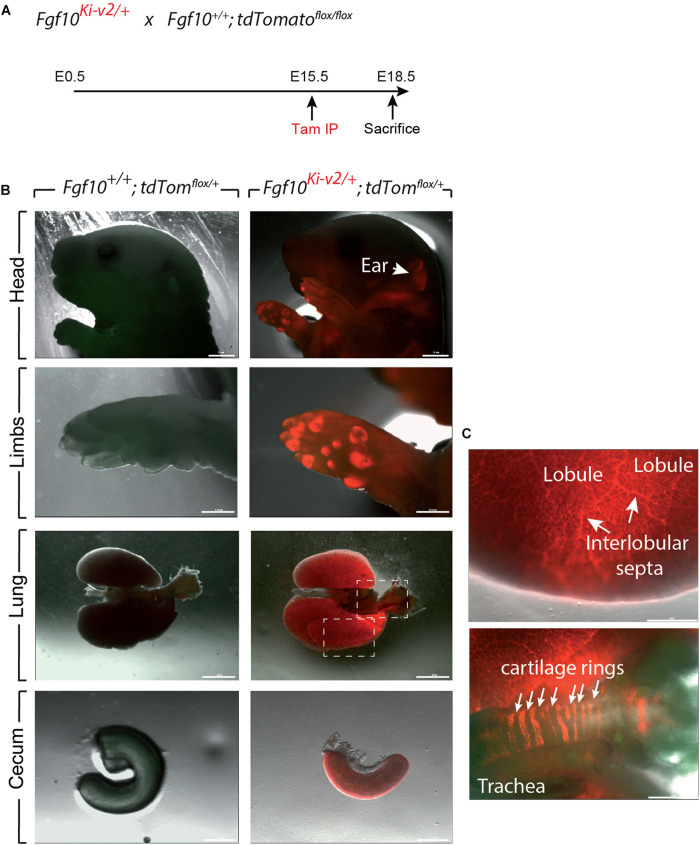
Validation of the labeling of FGF10^Pos^ cells at E15.5. **(A)** *Fgf10^*Ki-v*2/+^* were crossed with *Fgf10^+/+^*; *tdTom*^*flox*/*flox*^ mice. Pregnant females received a single IP injection of tamoxifen when the embryos were at E15.5 and sacrificed at E18.5. **(B)** Head, limbs, lung and cecum of *Fgf10^+/+^*; *tdTom^*flox*/+^* and *Fgf10^*Ki-v*2/+^*; *tdTom^*flox*/+^* embryos are shown. Note the expression in the external ear (arrow). **(C)** Higher magnification of lung and trachea showing enriched tdTomato expression in the interlobular septa and the presence of tdTomato expression between the cartilage rings, respectively. Scale bar in **(B)**: head: 1.5 mm, Limb: 0.75 mm, Lung: 0.5 mm, Cecum: 0.1 mm. Scale bar in **(C)**: Lung 25 μm, Trachea 125 μm.

In order to test the recombinase activity of Cre-ERT2, *Fgf10*^*Ki*–*v*2/+^ heterozygous mice were crossed with *tdTomato*^*flox*/*flox*^ reporter mice. Pregnant mice received a single intraperitoneal (IP) injection of tamoxifen at E11.5 (Figure 3A) or E15.5 (Figure 4A). Embryos were harvested at E18.5. No fluorescent signal was observed in *Fgf10^+/+^; tdTomato^*flox*/+^* embryos (Figures 3B, 4B; *n* = 4) indicating absence of recombination in control embryos and lack of leakiness of the *tdTomato*^*flox*^ allele. By contrast, tamoxifen treatment at E11.5 led to a strong fluorescent signal in the limbs, stomach, cecum, colon and lungs of *Fgf10^*Ki*–*v*2/+^; tdTomato^*flox*/+^* embryos (*n* = 3). In the limb, the labeled cells were more abundant in the digit tip area, known to express high level of *Fgf10* (Danopoulos et al., 2013). Along the gastro-intestinal tract, labeled cells were located in the anterior part of the stomach as well as in duodenum (data not shown) which are both reported to express high level of *Fgf10* (Lv et al., 2019). A similar observation was made in the cecum and the distal colon (Lv et al., 2019). Throughout the lung, we found a robust tdTomato expression with a higher expression in the interlobular septa. This is similar to what was observed with the previously validated *Fgf10^*LacZ*^* reporter line and *Fgf10^*KI*–*v*1/+^* line (Mailleux et al., 2005; El Agha et al., 2012, 2014). Interestingly, in the trachea, no labeled cells were observed in this experimental condition (Figure 3C). Additionally, tamoxifen treatment at E15.5 revealed strong fluorescent signal in the pinna of the developing ear as well as in the trachea and in between the cartilage rings (Figure 4C). These two additional expression domains are consistent with sites of *Fgf10* expression (Sala et al., 2011; Zhang et al., 2020). We therefore conclude that Cre expression reflects *Fgf10* expression and that this line can be used to target FGF10^Pos^ cells.

### FGF10^Pos^ Cells Labeled After Birth Contribute to the Lipofibroblast Lineage but Not to the Smooth Muscle Cell Lineage

**FIGURE 5:**
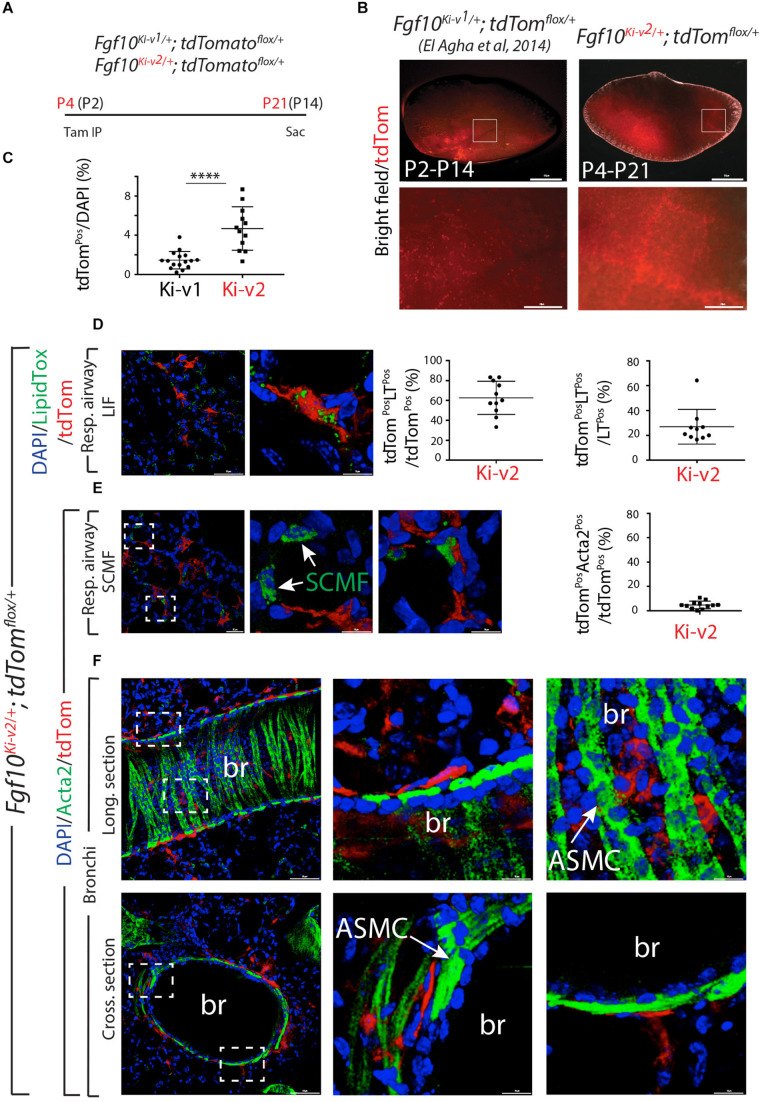
FGF10^Pos^ cells labeled after birth do not contribute significantly to secondary crest myofibroblasts during alveologenesis. **(A)** FGF10^Pos^ cells in *Fgf10^*Ki-v*2/+^; tdTomato^*flox*/+^* pups were labeled *in vivo* at P4 and analyzed at P21. We also used previously generated *Fgf10^*Ki-v*1/+^; tdTomato^*flox*/+^* samples labeled between P2 and P14. **(B)** Whole-mount fluorescence images of *Fgf10^*Ki-v*2/+^*; *tdTom^*flox*/+^* and *Fgf10^*Ki-v*1/+^*; *tdTom^*flox*/+^* lungs showing more abundant labeled cells in *Fgf10^*Ki-v*2/+^* vs. *Fgf10^*Ki-v*1/+^* lungs. **(C)** ACTA2 IF on *Fgf10^*Ki-v*2/+^*; *tdTom^*flox*/+^* lungs shows little contribution of FGF10^Pos^ cells to SCMF (ACTA2^Pos^tdTom^Pos^/ACTA2^Pos^). **(D)** Quantification of tdTom^Pos^ cells. Br: bronchi. Scale bar in **(B)**: low magnification: 0.5 mm, High magnification: 50 μm. Scale bar in **(D)**: low magnification: 50 μm, High magnification: 10 μm. Scale bar in **(E)**: Llow magnification: 25 μm, High magnification: 10 μm. Scale bar in **(F)**: Low magnification: 50 μm, High magnification: 10 μm. ****P ≤ 0.0001.

Using the previously generated *Fgf10^*Ki*–*v*1/+^* line, we demonstrated that FGF10^Pos^ cells labeled postnatally strongly contribute to the lipofibroblast (LIF) lineage but not the smooth muscle cell (SMC) lineage. In particular, they do not contribute in a major way to the ACTA2^Pos^ secondary crest myofibroblasts (SCMF) which are abundant during the first 2–3 weeks during alveologenesis which takes place from postnatal day 5 (P5) to -P28 (El Agha et al., 2014). To confirm this observation with the new *Fgf10*^*Ki*–*v*2/+^ line, we labeled FGF10^Pos^ cells at P4 and examined the status of the labeled cells at P21, 1 week before the end of the alveologenesis phase (Figure 5A). Analysis of the whole lung by fluorescence stereomicroscopy indicated a much higher number of labeled cells in the *Fgf10^*Ki*–*v*2^; tdTomato^*flox*/+^* lung compared with the *Fgf10^*Ki*–*v*1;^ tdTomato^*flox*/+^* lung (Figure 5B). Quantification of tdTom^Pos^ cells indicated that a higher percentile of tdTom^Pos^/DAPI is observed on sections of *Fgf10^*Ki*–*v*2^; tdTomato^*flox*/+^* vs. *Fgf10^*Ki*–*v*1;^ tdTomato^*flox*/+^* (4.7% ± 0.6% vs. 1.5% ± 0.2%, *n* = 2) thereby confirming the fluorescence stereomicroscopy results (Figure 5C). LipidTOX staining of these lungs was used to visualize LIFs (Figure 5D). Quantification of this staining indicated that 62.6% ± 5.0% (*n* = 2) of the total tdTom^Pos^ are LT^Pos^ and that 27.0% ± 4.4% (*n* = 2) of the LT^Pos^ derive from tdTom^Pos^ cells. These data are in line with our results obtained with the previous *Fgf10^*Ki*–*v*1/+^* line. Immunofluorescence (IF) for ACTA2 on these lungs was also carried out (Figures 5E,F). First, we quantified the number of ACTA2^Pos^/tdTom^Pos^ present in the respiratory airway (Figure 5E) and alveolar space (Supplementary Figure 2). ACTA2^Pos^ cells in the respiratory airway during alveologenesis mark secondary crest myofibroblasts (SCMF). We found 4.7% ± 0.9% (*n* = 2) tdTom^Pos^ACTA2^Pos^/tdTom^Pos^ indicating in our experimental conditions, a minimal commitment of the FGF10^Pos^ cells to the SCMF lineage. Second, we identified peribronchial tdTom^Pos^ cells both in longitudinal and cross sections of the bronchi (Figure 5F). Airway smooth muscle cells express ACTA2, display the typical bundle-like circular shape and are located in close proximity to the bronchial epithelium. tdTom^Pos^ cells are located close to ACTA2^Pos^-ASMCs but are nevertheless negative for ACTA2. A similar observation was made for the perivascular tdTom^Pos^ cells (data not shown).

### The New *Fgf10*^*Ki*–*v*2^ Line Allows More Efficient Labeling of FGF10^Pos^ Cells in the Adult Lung Compared With the Previous *Fgf10*^*KI*–*v*1^ Line

**FIGURE 6:**
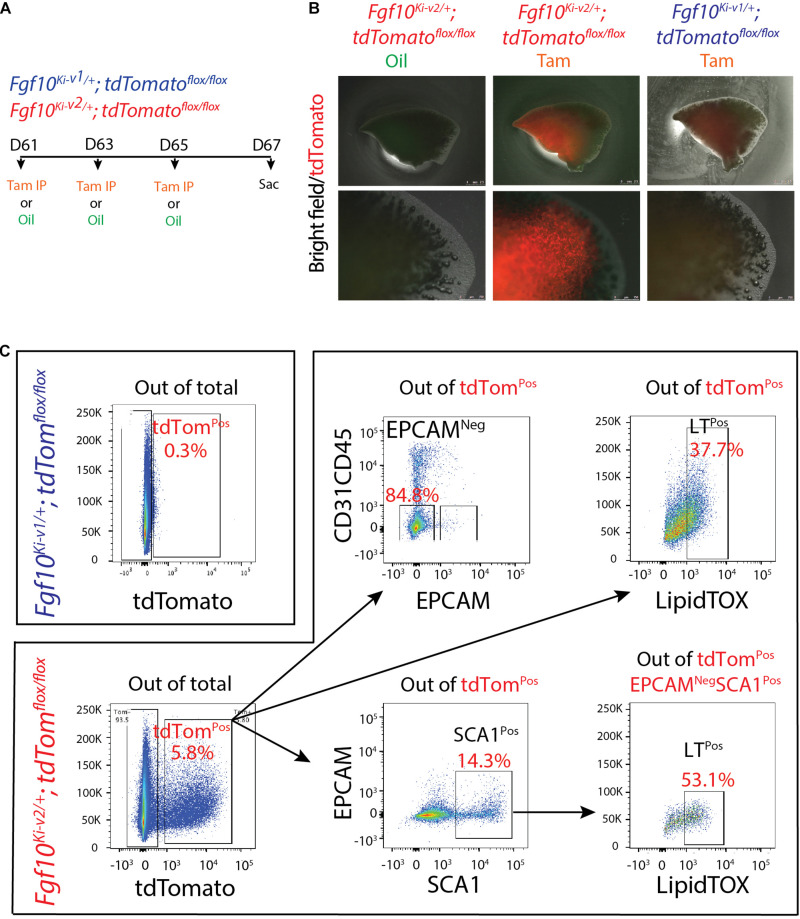
*Fgf10^*Ki-v*2/+^*; *tdTom^*flox*/+^* adult lungs display enhanced number of tdTom^Pos^ cells compared with to the *Fgf10^*Ki-v*1/+^*; *tdTom^*flox*/+^* adult lungs. **(A)** 2-months-old; *Fgf10^*Ki-v*1/+^*; *tdTom^*flox*/+^* and *Fgf10^*Ki-v*2/+^*; *tdTom^*flox*/+^* mice received 3 Tam IPs or oil at P61, P63 and P65 and were sacrificed at P67. **(B)** Whole-mount fluorescence images of oil-treated *Fgf10^*Ki-v*2/+^*; *tdTom^*flox*/+^*, Tam-treated *Fgf10^*Ki-v*2/+^*; *tdTom^*flox*/+^ and* Tam-treated *Fgf10^*Ki-v*1/+^*; *tdTom^*flox*/+^* lungs at P67. Higher magnification of the lungs are shown in the lower panel. **(C)** Flow cytometry analysis of *Fgf10^*Ki-v*2/+^*; *tdTom*^*flox*/*flox*^ lung homogenate. Scale bar in **(B)**: low magnification: 2.5 mm, High magnification: 0.75 mm.

Two months old *Fgf10*^*K*−*v*2^^/+^; *tdTomato^*flox*/*flox*^* mice were treated with Tam IP or oil at day 1 (D61), 3 (D63), and 5(D65) and the lungs were collected at day 7 (D67) (Figure 6A). No fluorescent signal was observed in oil-treated *Fgf10*^*K*−*v*2^^/+^; *tdTomato^*flox*/*flox*^* mice, indicating that the line is not leaky. By contrast, a solid signal was found in Tam-treated *Fgf10*^*Ki*–*v*2^^/+^; *tdTomato^*flox*/*flox*^* lungs. A weak signal was detected in Tam-treated *Fgf10*^*Ki*–*v*1^^/+^; *tdTomato^*flox*/*flox*^* lungs as described in a previously study (El Agha et al., 2014; Figure 6B). Flow cytometry analysis was also conducted to quantify the total number of tdTom^Pos^ cells in both conditions as well as their identity (El Agha et al., 2014; Figure 6C). Only 0.3% tdTom^Pos^ cells over total number of cells were detected in *Fgf10*^*Ki*–*v*1^^/+^; *tdTomato^*flox*/*flox*^.* This number is in line with the previously reported 0.1% (El Agha et al., 2014) and confirms that the *Fgf10*^*Ki*–*v*1^^/+^ line is not efficient to target FGF10^Pos^ cells in the adult lung. By contrast, we observed 5.8% of tdTom^Pos^ cells over total cells in *Fgf10*^*Ki*–*v*2^^/+^ lungs. Further analysis showed that these cells were mostly CD31^Neg^CD45^Neg^EPCAM^Neg^ cells (85%) identifying them as resident mesenchymal cells (rMC). 14.3% of the tdTom^Pos^ cells were also SCA1^High^, a functional marker of the rMC subpopulation capable of sustaining the self-renewal of AT2 stem cells in the alveolosphere organoid model (Taghizadeh et al., 2021). Of note 53% of the SCA1^High^ were LipidTOX^Pos^ identifying them as lipofibroblasts (LIFs). Altogether these results suggest that tdTom^Pos^ cells are heterogeneous and comprise a significant percentile of LIFs as previously reported (El Agha et al., 2014).

### The 3′UTR Region of the *Fgf10* Gene Contains Many Key Transcription Factor Binding Sites

**FIGURE 7:**
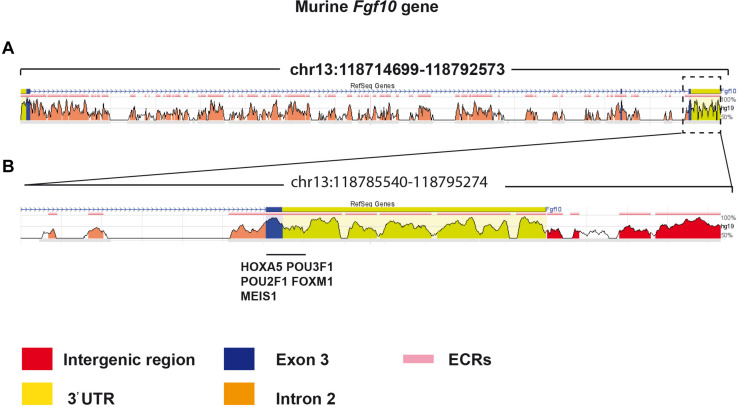
Bioinformatic analysis identified potential transcriptional factors binding sites in the 3′UTR of Exon 3. **(A)** The murine *Fgf10* gene is made of 3 exons. **(B)** High magnification of Exon 3 and the associated 3′UTR. Several putative important transcriptional factor binding sites were found in this region.

The decrease in *Fgf10* expression in *Fg10*^*Ki*–*v*2^ mice (Figure 2D) suggested that important transcription factor binding sites (TFBS) were impacted by the genetic manipulation in the 3′UTR of the *Fgf10* gene. We determined the identity of TFBS located at proximity of the 3′UTR of the *Fgf10* gene using an online TFBS prediction tool. We compared these TFBS with previously published TF expressed in the lung mesenchyme (Herriges et al., 2012). We found several key TFBS matching the previously reported TF expression in the lung such as *Hoxa5*, *Pou3f1*, *Pou2f1*, *Foxm1*, and *Meis1* (Figure 7). Interestingly, all these transcription factors appear to play a functional role in the lung. Mutant *Hoxa5* mice display decreased surfactant production and disrupted tracheal cartilage, leading to respiratory distress and low survival rate at birth (Aubin et al., 1997; Kinkead et al., 2004; Mandeville et al., 2006). *Pou3F1*, also known as *Oct6* is primarily expressed in neural cells. *Pou3F1* deletion caused lethality at birth due to respiratory distress (Bermingham et al., 1996, 2002; Ghazvini et al., 2002). The deletion of the other related transcription factor, *Pou2f1*, is associated with smaller body size of embryos and full lethality at birth (Wang et al., 2004). *Foxm1* expression plays a crucial role in both the epithelium and the mesenchyme. Conditional inactivation of *Foxm1* in the lung mesenchyme leads to increased smooth muscles around the proximal airways and reduced pulmonary microvasculature (Kim et al., 2005). In the lung epithelium, *Foxm1* conditional inactivation causes reduction in sacculation and delayed differentiation of alveolar epithelial type I cells (Kalin et al., 2008). Knockout of *Meis1* caused lethality during the embryonic stage around E14.5 due to microvascular and hematopoietic defects in the lung (Hisa et al., 2004).

## Discussion

FGF10 is an essential morphogen underlying the developmental process of multiple organs including the lung. FGF10 signaling is also crucial during homeostasis and in the process of injury/repair in the adult lung. *FGF10* dysregulation in human has been implicated in some major respiratory diseases, such as bronchopulmonary dysplasia (BPD), Idiopathic pulmonary fibrosis (IPF) and chronic obstructive pulmonary disease (COPD) (Yuan et al., 2018). For example, increased FGF10 expression level in IPF patients has been found (El Agha et al., 2017). However, FGF10 expression is inversely correlated to the disease progression with higher levels in stable IPF vs. lower level in end-stage IPF. Higher FGF10 expression in the early, stable stage of IPF is most likely correlated with the repair process. Insufficient FGF10 level in prematurely newborn infants is associated with arrested lung development at the saccular stage (Prince, 2018). *Fgf10* deficiency in a newborn mouse model of hyperoxia-induced BPD led to drastic increase in lethality associated with abnormal alveolar epithelial type 2 (AT2) cell differentiation as well as surfactant production (Chao et al., 2017).

FGF10 also performs a key function for the repair of the bronchial epithelium after injury (Volckaert et al., 2011). Our knowledge about the sources of FGF10 in this context has been evolving. FGF10 was first described to be expressed by airway smooth muscle cells (ASMCs) (Volckaert et al., 2011), whereas more recent work identified a peribronchiolar mesenchymal population capable of producing FGF10 during the repair process, which is not derived from the ASMCs (Moiseenko et al., 2020).

In COPD, the conducting airway epithelium undergoes massive remodeling causing an irreversible airway obstruction (Decramer et al., 2012). Interestingly, we have reported that the FGF10-HIPPO epithelial mesenchymal crosstalk also maintains and recruits lung basal stem cells in the conducting airways (Volckaert et al., 2017). While transient *Fgf10* expression by ASMCs is critical for proper airway epithelial regeneration in response to injury, sustained FGF10 secretion by the ASMC niche, in response to chronic ILK/HIPPO inactivation, results in pathological changes in airway architecture resembling the abnormalities seen in COPD. The inhibition of FGF10/FGFR2b signaling may therefore be an interesting approach to treat chronic obstructive airway lung diseases. Conversely, the opposite situation might occur in the respiratory airways in that destruction of the alveolar compartments resulting in emphysema may be due to insufficient FGF signaling. Interestingly, recombinant FGF7 has been reported to induce *de novo*-alveologenesis in the elastase model of emphysema in mice (Yildirim et al., 2010).

The previous *Fgf10*^*Ki*–*V*1^ model was mainly used to trace the FGF10^Pos^ cells during embryonic development. A near complete loss of the labeling capacity of FGF10^Pos^ cells during postnatal stages limited its utilization in the analysis of their cell fate in adult lung homeostasis and during the process of injury/repair. In order to overcome the limitations of the *Fgf10*^*Ki*–*V*1^ line, we generated and validated this new knock-in *Fgf10*^*Ki*–*V*2^ line. Upon crossing with a *tdTomato* reporter line, we demonstrated that the tdTomato expression domain faithfully reproduced the previously reported the *Fgf10* expression pattern (El Agha et al., 2012), and a more robust labeling of FGF10^Pos^ cells was achieved in the postnatal stages in spite of a mismatch between *Cre* and *Fgf10* expression, which could be explained by the disruption of critical TFBS located in the 3′UTR of the *Fgf10* gene. Therefore, this line will be a valuable tool to further define mesenchymal cell populations in the adult lung contributing to the repair process after injury. Combined crosses with existing or novel Dre-ERT2 recombinase driver lines may allow to capture subpopulations of FGF10^Pos^ cells/lineages based on the expression of two markers (Jones et al., 2019). The main FGF10^Pos^ subpopulation is represented by the lipid-containing alveolar interstitial fibroblasts (lipofibroblasts or LIFs). More and more studies have acknowledged LIFs as an essential piece of the AT2 stem cell niche in the rodent lungs. Despite the fact that LIFs were initially believed to only assist AT2 cells in surfactant production during neonatal life, recent studies have shown that these cells are important for self-renewal and differentiation of AT2 stem cells during adulthood (Barkauskas et al., 2013). In spite of the increasing interests in lipofibroblast biology, little is known about their cellular origin or the molecular pathways that control their formation during embryonic development. We have shown that in the developing mouse lung, FGF10^Pos^ cells labeled at E11.5 or E15.5 are progenitors for LIFs (El Agha et al., 2014). In addition, FGF10 is also essential for the differentiation of these progenitors into the LIF lineage (Al Alam et al., 2015). We have also reported the existence of FGF10^Pos^-LIF as well as FGF10^Neg^-LIFs (Al Alam et al., 2015). The difference between these two populations is still unclear and will require further studies. In the context of bleomycin-induced lung fibrosis, *in vivo* lineage tracing indicates that LIFs transdifferentiate into activated myofibroblast during fibrosis formation and that a significant proportion of the labeled activated myofibroblasts transdifferentiate back to LIFs during fibrosis resolution (El Agha et al., 2017).

In conclusion, we have successfully generated a new *Fgf10*^*Cre*–*ERT*2^ line with enhanced labeling efficiency of FGF10^Pos^ cells postnatally. This line, which displays normal expression of *Fgf10* in *Fgf10^*Cre*–*ERT*2/+^*, avoids many developmental defects linked to deficient *Fgf10* expression. Therefore, it paves the way for performing cell-autonomous based studies to investigate the role of these FGF10^Pos^ cells as well as associated signaling pathways during lung development and disease.

## Data Availability Statement

The original contributions presented in the study are included in the article/Supplementary Material, further inquiries can be directed to the corresponding author/s.

## Ethics Statement

Animal experiments were reviewed and approved by the Regierungspraesidium Giessen (approval number RP GI/47- 2019).

## Author Contributions

XC, ST, AIV-A, and LC performed the experiments. SH, CC, and J-SZ contributed to methodology. EEA and SB conceived the study. XC and SB wrote the manuscript. J-SZ, EEA, and SB edited the manuscript. All authors contributed to the article and approved the submitted version.

## Conflict of Interest

The authors declare that the research was conducted in the absence of any commercial or financial relationships that could be construed as a potential conflict of interest.

## Footnotes

1 https://www.ncbi.nlm.nih.gov/tools/primer-blast/

2 http://alggen.lsi.upc.edu/recerca/menu_recerca.html

## Supplementary Material

Supplementary Figure 1 Comparative *Fgf10* expression in *Fgf10^+/+^ and Fgf10^*Ki*–*v*2/+^ lungs vs. Fgf10*^*Ki*–*v*2/*Ki*–*v*2^ embryos at E15.5. qPCR was used to determine *Fgf10* expression.

Supplementary Figure 2 Co-expression of ACTA2 and tdTomato in FGF10^Pos^ cells. *Fgf10^*Ki*–*v*1^; tdTomato^*flox*/+^* pups received one injection of Tam subcutaneously at P2 and were analyzed at P14.
