# Supplementary figures and images for: Validation of a Novel Fgf10Cre–ERT2 Knock-in Mouse Line Targeting FGF10Pos Cells Postnatally
Source: Front Cell Dev Biol. 2021 May 13;9:671841. doi: 10.3389/fcell.2021.671841 (PMC8155496; doi:10.3389/fcell.2021.671841)

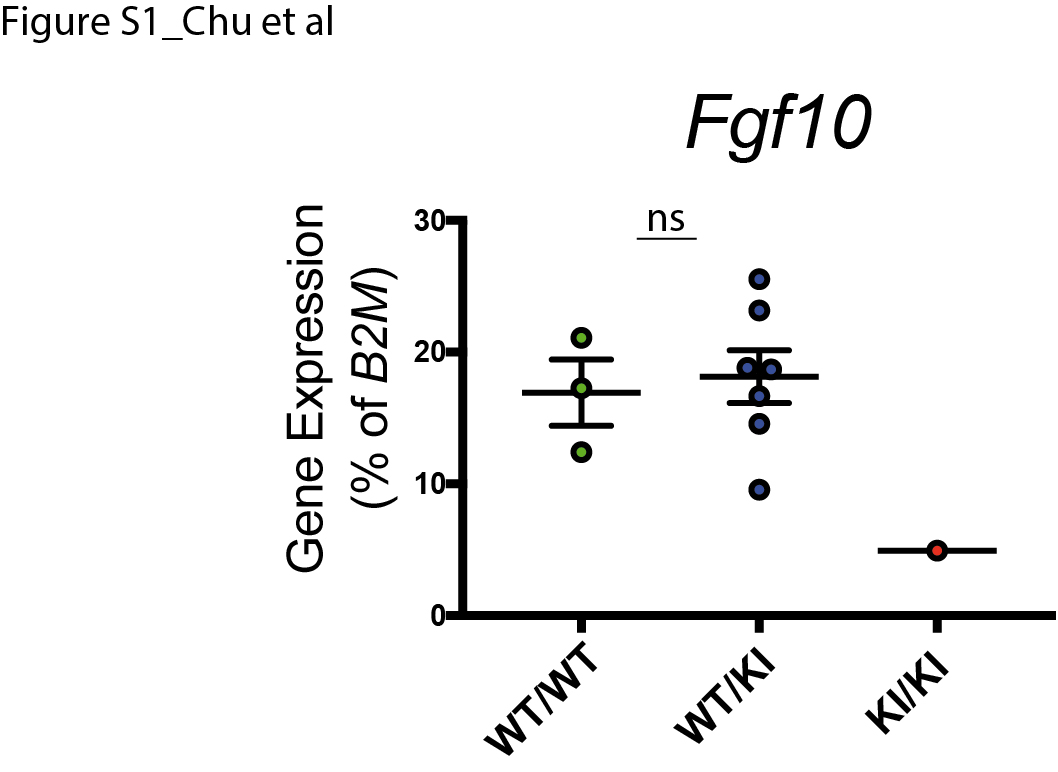

Supplement: Supplementary Figure 1 — Comparative Fgf10 expression in Fgf10+/+ and Fgf10Ki–v2/+ lungs vs. Fgf10Ki–v2/Ki–v2 embryos at E15.5. qPCR was used to determine Fgf10 expression. [file Image_1.JPEG]

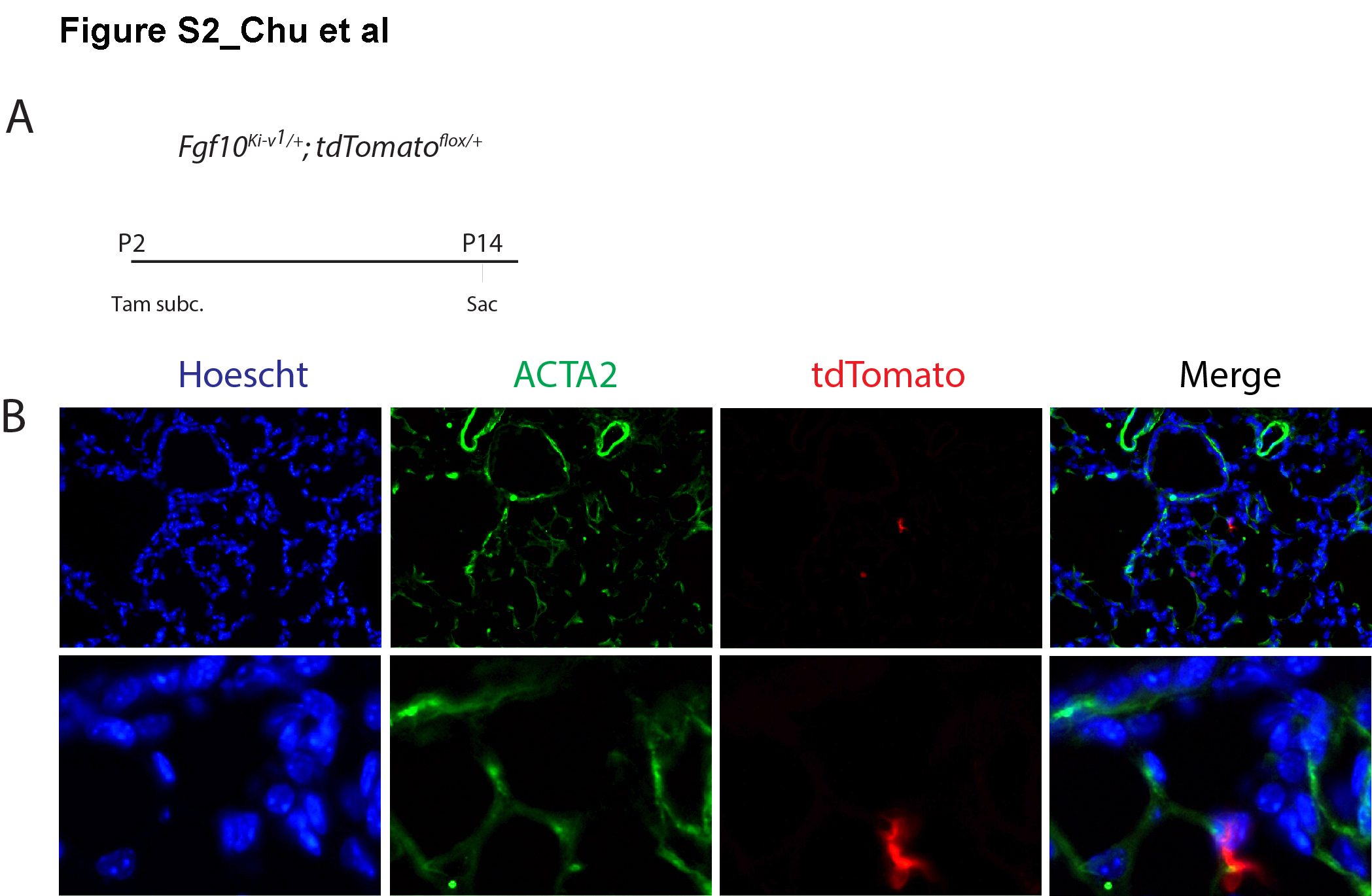

Supplement: Supplementary Figure 2 — Co-expression of ACTA2 and tdTomato in FGF10Pos cells. Fgf10Ki–v1; tdTomatoflox/+ pups received one injection of Tam subcutaneously at P2 and were analyzed at P14. [file Image_2.JPEG]
