# Supplementary material for: Validation of a Novel Fgf10Cre–ERT2 Knock-in Mouse Line Targeting FGF10Pos Cells Postnatally
Source: Front Cell Dev Biol. 2021 May 13;9:671841. doi: 10.3389/fcell.2021.671841 (PMC8155496; doi:10.3389/fcell.2021.671841)
Supplement: Supplementary file 3 [file Table_1.DOCX]

**TABLE S1**

| **Genes** |  | **Primers** | **UPL Probes** |
| --- | --- | --- | --- |
| ***Fgf10* (P1&P2)** | **Forward** | 5’-AACACCTCTGCTCACTTCCTC-3’ | 251 |
|  | **Reverse** | 5’-AGGGTCCACCTTCCGCTTTT-3’ |  |
| ***Fgf10* (P3&P4)** | **Forward** | 5’-GCAGGCAAATGTATGTGGCA-3’ | 580 |
|  | **Reverse** | 5’-TGCTTGCGTGTCTTACTGCT-3’ |  |
| ***Cre*** | **Forward** | 5’-CGCAAGAACCTGATGGACATG-3’ | 82 |
|  | **Reverse** | 5’-ACCGGCAAACGGACAGAA-3’ |  |
| ***β2-Microglobulin(B2M)*** | **Forward** | 5’-CTCACACTGAATTCACCCCC -3’ | 54 |
|  | **Reverse** | 5’-GCTTAACTCTGCAGGCGTAT-3’ |  |
